# Pembrolizumab in Practice: Assessing Safety and Effectiveness Through a Retrospective Frame

**DOI:** 10.7759/cureus.101787

**Published:** 2026-01-18

**Authors:** Shalini Thomas, Princy Palatty, Laxmi Govindraj, Visalakshi Potukuchi

**Affiliations:** 1 Department of Pharmacology, Amrita Institute of Medical Science and Research Center, Amrita Vishwa Vidhyapeetham, Ernakulam, IND; 2 Department of Medicine, Amrita Institute of Medical Science and Research Center, Amrita Vishwa Vidhyapeetham, Ernakulam, IND

**Keywords:** adverse reaction, carcinoma breast, effectiveness, hypothyroidism, immunotherapy, lenvatinib, oncology, pd -1 inhibitor, pembrolizumab, renal cell cancer

## Abstract

Introduction

Immune evasion is one of the surest methods to initiate an effective antitumour response. Programmed cell death protein 1 (PD-1) is a highly expressed immune checkpoint receptor on lymphocytes and plays an important role in regulating T-cell responses to reduce damage to surrounding normal tissues. This methodology has been successfully tried in metastatic non-small-cell lung cancer (NSCLC), renal cell cancer, urothelial cancer, bladder cancer, colon cancer, etc.

Study aim

The aim of this study was to determine the effectiveness of pembrolizumab in various cancers over the last five years and to analyse the pattern of Adverse Drug Reactions (ADR) in patients treated with pembrolizumab.

Methodology

This is a retrospective study with patients who received at least one dose of pembrolizumab from August 31st, 2019, to July 31st, 2024. Demographic data, details of pembrolizumab therapy, documented adverse reactions, and patient response as per RECIST (Response Evaluation Criteria in Solid Tumours) criteria were noted.

Results

A total of 111 patients treated with pembrolizumab across 22 cancer types received 1,238 treatment cycles (mean 11 cycles per patient). Pembrolizumab demonstrated meaningful clinical effectiveness, with an objective response rate (ORR) = 14.5% and a disease control rate (DCR) is 61.3% across a heterogeneous cancer cohort. Among individual malignancies, breast cancer patients showed the most favorable outcomes, with 33.3% achieving complete or partial response and 55.6% maintaining stable disease.

Adverse events, including 21 immune-mediated events, were observed in 80.2% of patients. The majority of adverse reactions were mild to moderate (Grade 1-2). Five patients developed Grade 3 thrombocytopenia, all of whom were successfully rechallenged. Concomitant lenvatinib therapy was significantly associated with increased toxicity (p=0.041), particularly thyroid dysfunction. Notably, patients who developed adverse reactions demonstrated better clinical outcomes than those without toxicity.

Conclusion

Pembrolizumab demonstrated meaningful effectiveness with an ORR of 14.5%, a disease control rate of 61.3%, and a generally favorable safety profile, with most adverse reactions being mild to moderate and immune-related toxicities manageable. Increased vigilance is required when combined with lenvatinib due to higher thyroid dysfunction risk. Overall, pembrolizumab remains an effective and well-tolerated option across diverse cancers, underscoring the importance of careful patient selection and close monitoring to optimize therapeutic outcomes.

## Introduction

Pembrolizumab is a humanized monoclonal antibody targeting the programmed death-1 (PD-1) receptor that enhances antitumor immune responses and has demonstrated significant clinical efficacy with an acceptable safety profile across multiple malignancies [1]. It binds with high affinity to the PD-1 receptor on activated T lymphocytes, thereby blocking its interaction with the ligands PD-L1 and PD-L2, which are frequently overexpressed on tumor cells to evade immune surveillance [2]. By disrupting this inhibitory pathway, pembrolizumab effectively releases the immunological “brakes” on T cells, restoring their capacity to proliferate, secrete cytokines, and induce apoptosis in malignant cells [3]. Consequently, this immune reinvigoration transforms the tumor microenvironment from an immunosuppressive state to an immunostimulatory one, thereby enhancing antitumor immune responses [4]. Notably, pembrolizumab, like other PD-1-targeting antibodies, utilizes an immunoglobulin (Ig)G4 backbone to limit Fc-mediated effector functions and avoid depletion of activated T cells [5].

Clinically, pembrolizumab has demonstrated durable tumor control and improved survival outcomes in several malignancies, including melanoma, renal cell carcinoma, non-small cell lung cancer, breast carcinoma, head and neck cancers, and urothelial carcinoma, among others [6]. However, despite these therapeutic benefits, immune activation induced by pembrolizumab may also precipitate immune-related adverse events. These toxicities necessitate vigilant monitoring and timely management to optimize both safety and treatment effectiveness in diverse patient populations. Against this background, the present study aims to evaluate the effectiveness of pembrolizumab across various cancer types and to analyze the pattern of adverse drug reactions observed in patients treated with pembrolizumab over the past five years.

## Materials and methods

Study population and design

This study was designed as a retrospective, observational cohort study conducted at the Amrita Institute of Medical Science, Kochi, Kerala, India, a tertiary care center, and evaluated the safety and effectiveness of pembrolizumab in routine clinical practice. Pembrolizumab dosing is 200 mg intravenous (IV) every three weeks or 400 mg IV every six weeks (infused over 30-60 minutes depending on cancer type schedule). A waiver of consent was granted by the Institutional Ethics Committee (IEC). Data for all patients who received at least one dose of pembrolizumab in a five-year duration, i.e., between August 1, 2019, and July 31, 2024, were retrieved from medical records. The study lasted two months.

Inclusion and exclusion criteria

Adult patients aged ≥18 years were eligible for inclusion. Patients were required to have a histologically or cytologically confirmed malignancy for which pembrolizumab was prescribed according to standard clinical indications. Eligible patients must have received at least one dose of pembrolizumab, either as monotherapy or in combination with other anticancer agents. Patients treated in any therapeutic setting, including adjuvant, neoadjuvant, first-line, or subsequent lines of therapy, were included. Only patients with adequate medical records documenting treatment details, adverse drug reactions, and treatment response, and who had undergone at least one follow-up clinical evaluation after drug administration, were considered for analysis. Patients with incomplete or missing clinical records, particularly those lacking data on adverse events or treatment outcomes, and those lost to follow-up immediately after the first dose, were excluded to ensure reliable safety and effectiveness assessment.

Data collection

Data were collected retrospectively from the Medical Records Department. Information was extracted from electronic medical records, including outpatient case files, inpatient records, chemotherapy administration logs, and relevant laboratory and radiological reports.

Statistical analysis

**Figure 1:**
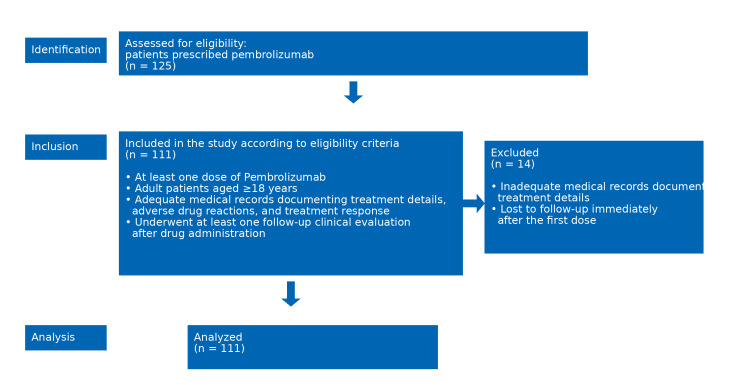
STROBE flow diagram of the study The STrengthening the Reporting of Observational Studies in Epidemiology (STROBE) diagram is illustrating the patient selection criteria and study cohort formation for the retrospective evaluation of the safety and effectiveness of pembrolizumab.

Statistical analysis was performed using SPSS Statistics software, version 20.0 (IBM Corp., Armonk, NY). Categorical variables were summarized as frequencies and percentages. The Pearson Chi-square test was used to assess the statistical significance of associations between categorical variables across study groups, with appropriate interpretation of results. Demographic characteristics, details of pembrolizumab therapy, observed adverse drug reactions, and patient responses, assessed according to the Response Evaluation Criteria in Solid Tumours (RECIST 1.1), were documented and analyzed (Figure 1).

## Results

**Figure 2:**
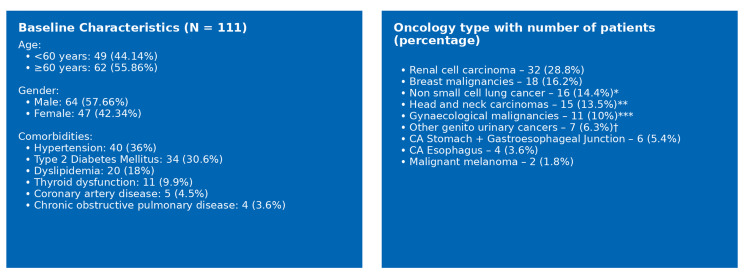
Baseline characteristics and oncology type distribution of patients on pembrolizumab included in this study *Non-small cell carcinoma (CA) includes Adenocarcinoma lung and Squamous cell carcinoma; **Head and neck carcinoma includes CA Alveolus, CA Hard Plate, CA Larynx, CA Oropharynx, CA Retromolar Trigone, CA Tongue, and  CA Hypopharynx; ***Gynaecological malignancies include CA Endometrium and CA Cervix; †Other genito-urinary cancers include CA Adrenal, Urothelial carcinoma, CA Ureter, CA Bladder.

A total of 111 patients who received at least one dose of pembrolizumab were included in the study, accounting for a cumulative 1,238 treatment cycles, with a mean of 11 cycles per patient. The median age of the cohort was 61 years. Among the study population, 49 patients (44.14%) were aged below 60 years, while 62 patients (55.86%) were aged 60 years or older. The cohort comprised 64 males (57.66%) and 47 females (42.34%), as depicted in Figure 2.

With regard to prior oncological treatments, the most common concomitant medications in the study population were platinum coordination complexes, used in 32 patients (28.83%), followed by microtubule-damaging agents (taxanes) in 18 patients (16.22%). Pembrolizumab was administered across 22 different cancer types (Figure 1). It was most frequently used in urogenital malignancies, particularly renal cell carcinoma, accounting for 32 patients (29%). This was followed by breast cancer in 18 patients (16.2%) and lung adenocarcinoma in 15 patients (13.5%). Regarding treatment regimens, 36 patients (32.43%) received pembrolizumab as monotherapy, whereas 75 patients (67.57%) were treated with pembrolizumab in combination with chemotherapy or targeted therapy.

Treatment response to pembrolizumab

**Figure 3:**
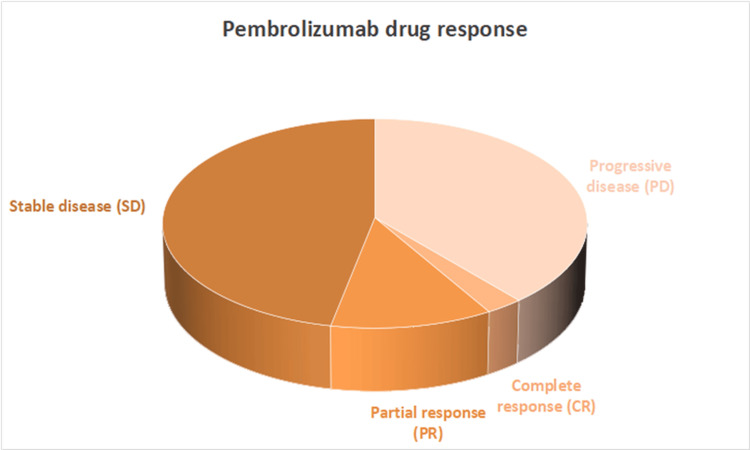
Distribution of treatment responses among patients receiving pembrolizumab includees regressing, partially regressing, stable, and progressive disease outcomes.

Treatment response analysis revealed that among the 111 patients, three patients (2.70%) achieved a complete response and 13 patients (11.71%) demonstrated a partial response. Stable disease was observed in 52 patients (46.85%), while 43 patients (38.74%) experienced tumor progression, as illustrated in Figure 2. Objective response rate (ORR) is 14.5%, and disease control rate (DCR) is 61.3% (Figure 3).

Response in monotherapy versus combination therapy

**Figure 4:**
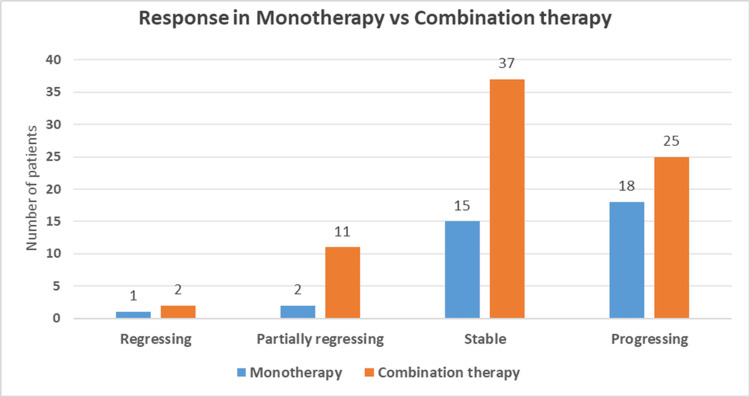
Distribution of treatment responses to pembrolizumab across cancer types: monotherapy vs combination therapy

In the monotherapy group, one patient (2.78%) demonstrated complete regression, while two patients (5.56%) showed partial regression. Stable disease was achieved in 15 patients (41.66%), whereas 18 patients (50%) experienced disease progression. In contrast, outcomes in the combination therapy group were comparatively more favorable. Complete regression was observed in two patients (2.67%), and partial regression in 11 patients (14.67%). Additionally, 37 patients (49.33%) achieved stable disease, while 25 patients (33.33%) experienced disease progression, as illustrated in Figure 4.

The most frequently used treatment regimen in the study population was carboplatin-paclitaxel, administered in 26 patients (23.4%). This combination was predominantly prescribed for breast cancer, followed by its use in carcinoma of the hypopharynx, lung adenocarcinoma, squamous cell carcinoma of the lung, carcinoma of the esophagus, carcinoma of the hard palate, endometrial cancer, carcinoma of the tongue, and carcinoma of the cervix. The second most common concomitant regimen was lenvatinib, which was used in 20 patients (18%), primarily in those with renal cell carcinoma, followed by patients with endometrial cancer. In addition to these regimens, several other combination therapies were employed less frequently, including carboplatin-pemetrexed in six patients (5.4%), carboplatin-docetaxel in six patients (5.4%), and axitinib in five patients (4.5%), as summarized in Table 1.

**Table 1:** Combination regimens administered with pembrolizumab across various cancer types * include other oncology regimens involving gemcitabine, cisplatin, capcitabine, trastuzumab, oxaliplatin, and denosumab. CA: Carcinoma.

Combination Regimens	Number of Patients	Percentage	Cancer Type
Carboplatin–Paclitaxel	26	23.4%	CA Breast
			CA Hypopharynx
			Adenocarcinoma Lung
			Squamous Cell Carcinoma Lung
			CA Oesophagus
			CA Hard Palate
			CA Endometrium
			CA Tongue
			CA Cervix
Lenvatinib	20	18%	Renal cell carcinoma
			CA Endometrium
Carboplatin–Pemetrexed	6	5.4%	Adenocarcinoma lung
Carboplatin–Docetaxel	6	5.4%	CA Breast
Axitinib	5	4.5%	Renal cell carcinoma
Others*	12	13.6%	Adenocarcinoma lung
			Renal carcinoma lung
			CA stomach
			CA GE Junction
			CA oesophagus
			Carcinoma Oropharynx
			CA Retro molar Trigone
			CA Breast
			CA Cervix
			CA Bladder

**Figure 5:**
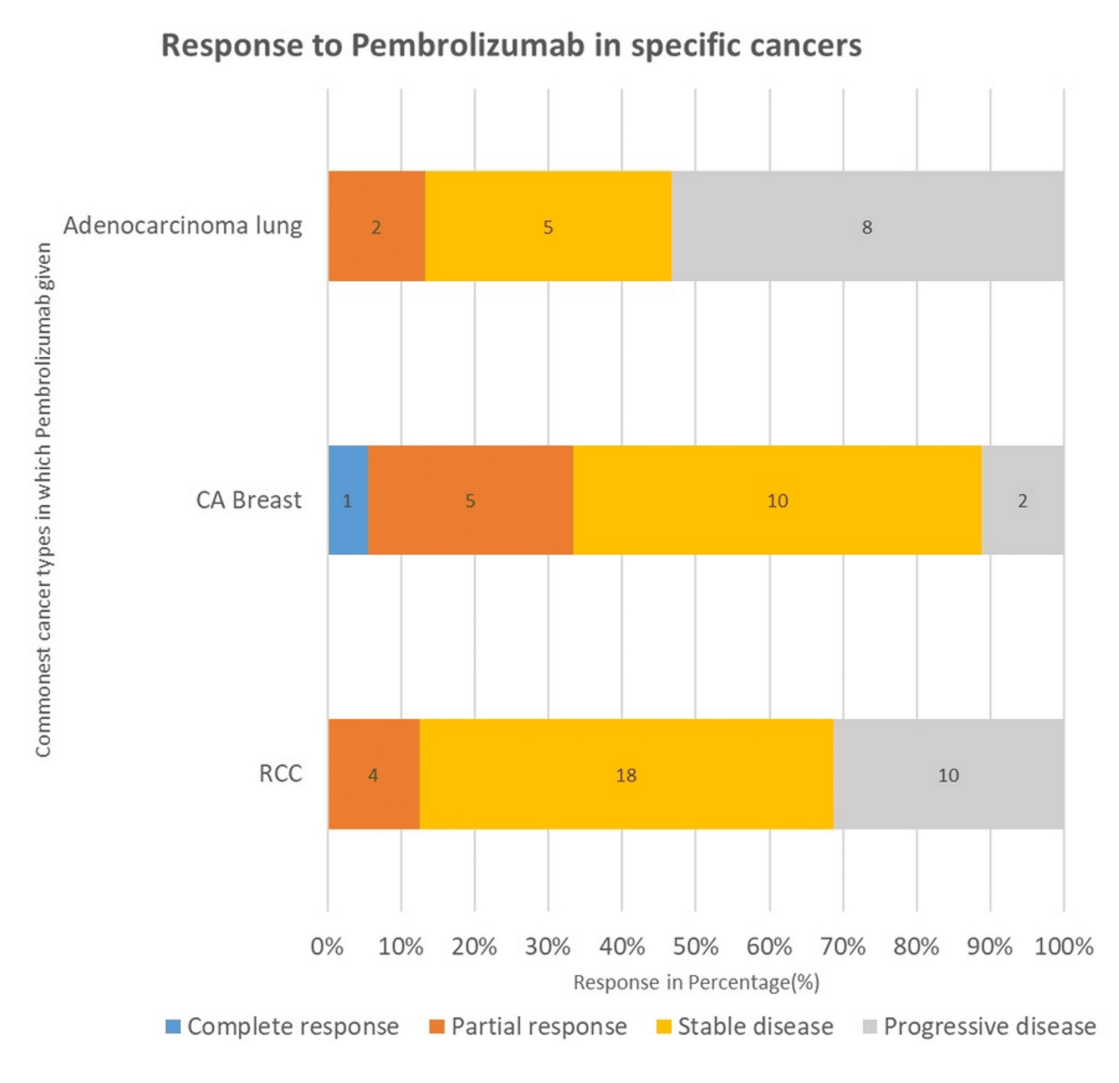
Treatment response to pembrolizumab across major cancer subgroups

Patients who received lenvatinib concomitantly with pembrolizumab demonstrated a favorable therapeutic profile. Among these patients, 12 (60%) achieved stable disease, four (20%) showed a partial response, and four (20%) experienced disease progression. Pembrolizumab was most frequently administered to patients with renal cell carcinoma, lung adenocarcinoma, and breast cancer. Consequently, treatment responses in these three malignancies were analyzed in greater detail and given particular emphasis in the present study, as illustrated in Figure 5.

Treatment response by cancer type

The most favorable treatment responses were observed among breast cancer patients, with six patients (33.34%) achieving a complete or partial response and 10 patients (55.56%) demonstrating stable disease. In contrast, patients with renal cell carcinoma predominantly exhibited stable disease, which was noted in 18 patients (56.25%). Among patients with lung adenocarcinoma, partial response was observed in two patients (13.33%), while stable disease was achieved in five patients (33.3%). Of the total 32 patients with renal cell carcinoma, pembrolizumab was administered as adjuvant therapy in 21 patients (65.63%), whereas the remaining 11 patients (34.38%) received pembrolizumab as first-line therapy.

Safety profile of pembrolizumab

A total of 89 patients (80.18%) experienced at least one adverse drug reaction, with a cumulative 201 adverse drug reactions reported across 1,238 treatment cycles. According to the Common Terminology Criteria for Adverse Events version 5.0, Grade 1 adverse drug reactions were observed in 74 patients (66.6%), Grade 2 in 32 patients (29%), and Grade 3 in five patients (4.5%). No Grade 4 adverse drug reactions were documented during the study period.

Among gastrointestinal adverse events, vomiting was reported in nine patients (8.11%), while constipation occurred in eight patients (7.21%). Generalized tiredness was the most frequently reported systemic adverse drug reaction, affecting 20 patients (18.02%). Cough was the most common respiratory adverse drug reaction, noted in 12 patients (10.81%), and itching was reported by 11 patients (9.91%). Vomiting was more commonly observed in patients receiving combination therapy, affecting nine patients (12%), compared with one patient (2.78%) in the monotherapy group. Immune-related adverse drug reactions observed in the study population are summarized in Table 2.

**Table 2:** Spectrum of immune-mediated adverse reactions associated with pembrolizumab therapy in the study population

Adverse Reactions	Number of patients (%)
Thyroiditis	8 (7.21%)
Neuropathic pain	6 (5.41%)
Severe thrombocytopenia	5 (4.5%)
Polyarthralgia	1 (0.9%)
Pneumonitis	1 (0.9%)

Among patients with renal cell carcinoma, adverse drug reactions were less frequent when pembrolizumab was administered in the adjuvant setting compared with its use as first-line therapy in metastatic disease (71.4% vs. 81.8%). As adverse reactions associated with the concomitant use of axitinib or lenvatinib are specifically highlighted in the Food and Drug Administration (FDA)-approved labeling for pembrolizumab, the present study included a focused evaluation of patients receiving these agents in combination. Adverse drug reactions were observed more frequently when lenvatinib was administered as a concomitant medication, occurring in 12 of 20 patients (p=0.041, Fisher’s exact test).

Among the 20 patients who received concomitant lenvatinib, 12 patients (60%) developed thyroid dysfunction as an adverse drug reaction (p<0.001, Fisher’s exact test), with an odds ratio of 21.2 (95% CI: 6.2-72). According to Common Terminology Criteria for Adverse Events version 5.0, the majority of these events were classified as Grade 2, and affected patients were initiated on thyroid hormone supplementation.

When treatment response was analyzed in relation to toxicity, among the 89 patients who experienced at least one adverse drug reaction, the most common outcome was stable disease, observed in 45 patients (50%). Complete response was noted in three patients (3.5%), partial response in 10 patients (11.6%), and progressive disease in 31 patients (34.9%). In contrast, among patients without adverse drug reactions (n=22), the majority experienced disease progression in 11 patients (52%), while stable disease was observed in eight patients (36%). No complete responses were recorded in this group.

## Discussion

The present study included 111 patients who received pembrolizumab across 22 different cancer types, with the highest representation observed in renal cell carcinoma, followed by non-small cell lung cancer and breast cancer. The cohort reflected the typical demographic profile of Indian oncology patients, with 44.1% aged below 60 years and 55.9% aged 60 years or older. Male patients constituted 57.66% of the study population, while females accounted for 44.4%.

In terms of efficacy, 68 patients (61%) experienced clinical benefit, including improvement in quality of life, whereas 43 patients (38.7%) demonstrated disease progression, indicating favorable therapeutic activity overall. These findings are comparable to those reported in the meta-analysis by Kaiheng Gao et al., which demonstrated significant tumor control and a manageable safety profile for pembrolizumab in non-small cell lung cancer patients [7].

ORR (complete response (CR)+ partial response (PR)) and DCR (CR, PR, and stable disease (SD) combined) observed in this study were 14.5% and 61.3% across various malignancies, which were higher than those reported in the above-mentioned meta-analysis. Although the proportion of patients achieving objective tumor shrinkage was modest, a substantial number experienced disease stabilization, reflecting meaningful clinical benefit in a heterogeneous real-world population. This disease control rate underscores pembrolizumab’s capacity to maintain tumor suppression even in the absence of radiological regression, consistent with the delayed and sustained immune-mediated responses characteristic of immune checkpoint inhibitors.

Consistent with findings reported by Dun-Chang et al., pembrolizumab-based combination regimens have been associated with improved antitumor efficacy compared with monotherapy in several malignancies [8]. The overall response rate was higher in the combination group (17.3% vs. 8.2%), with a greater proportion of patients achieving stable disease (49.3% vs. 41.6%) and a lower rate of disease progression (33.3% vs. 50%). These results suggest enhanced antitumor efficacy when pembrolizumab is administered alongside other therapeutic agents.

Improved efficacy with pembrolizumab-based combination regimens has been attributed to synergistic interactions that enhance immune activation and favorably modulate the tumor microenvironment [8,9]. Despite meaningful clinical benefit in a proportion of patients, others experienced disease progression, reflecting heterogeneity in response to pembrolizumab therapy [10]. In this cohort, the most commonly used pembrolizumab-based regimen was carboplatin-paclitaxel, predominantly in patients with breast cancer. Among patients receiving lenvatinib concomitantly with pembrolizumab, primarily those with renal cell carcinoma and endometrial cancer, 60% achieved stable disease, 20% demonstrated partial response, and 20% experienced disease progression, indicating pembrolizumab-based therapeutic strategies have demonstrated clinical activity in selected malignancies [11]. Pembrolizumab demonstrated notable efficacy in breast cancer patients (n=18), with 5.5% achieving complete response, 27.8% partial response, and 55.5% stable disease, representing the most favorable outcomes among the malignancies studied. Favorable responses were also observed in patients with renal cell carcinoma.

Regarding safety, grading according to the Common Terminology Criteria for Adverse Events (CTCAE) version 5.0 revealed that the majority of adverse drug reactions were mild, with Grade 1 reactions accounting for 66.6% and Grade 2 reactions for 29%. Only 4.5% of patients experienced Grade 3 toxicities, and no Grade 4 events were reported. This safety profile aligns with findings reported by Liu et al., pembrolizumab has been associated with a consistent, generally manageable safety profile across multiple advanced malignancies in clinical experience and pooled analyses of clinical trials [12].

The most commonly observed adverse drug reactions were fatigue (18.2%), cough (10.8%), vomiting (10%), constipation (9%), and itching (9.9%), consistent with findings from previous clinical trials and post-marketing surveillance data [13]. Vomiting was more frequently observed in the combination therapy group, likely attributable to concomitant chemotherapy or targeted agents. Overall, the low incidence of severe toxicities supports the favorable tolerability of Pembrolizumab compared with conventional chemotherapy.

Immune-related adverse events were less frequent but clinically significant and included thyroiditis (7.21%) [14], severe thrombocytopenia (4.50%), neuropathic events (5.41%), including one case of chronic inflammatory demyelinating polyneuropathy, as well as isolated cases of polyarthralgia and pneumonitis. Notably, patients with severe thrombocytopenia did not experience recurrence upon rechallenge, in contrast to findings reported by Perihan Perkin et al., in which, after managing thrombocytopenia with corticosteroids and with subsequent platelet recovery, rechallenging pembrolizumab led to recurrence of thrombocytopenia, prompting permanent discontinuation of pembrolizumab [15]. These observations further support pembrolizumab’s favorable risk-benefit profile while underscoring the importance of vigilant monitoring for rare immune-mediated toxicities.

Adverse drug reactions were less frequent in patients receiving pembrolizumab in the adjuvant setting (71.4%) compared with those treated in the first-line metastatic setting (81.8%), suggesting improved tolerability in the postoperative setting. None of the patients in the adjuvant group experienced adverse reactions greater than Grade 2, in contrast to findings reported by Mattigk et al., where 26% of renal cell carcinoma patients receiving adjuvant pembrolizumab developed Grade 3 or higher toxicities [16].

In agreement with findings reported by Bisschop et al., patients who developed immune-mediated adverse drug reactions demonstrated improved clinical outcomes [17]. In this study, 50% of such patients achieved stable disease, and 3.5% achieved complete response, whereas patients without adverse drug reactions experienced a higher rate of disease progression (52%). This association likely reflects heightened immune activation and systemic drug exposure, suggesting that immune-mediated toxicities may serve as surrogate markers of therapeutic efficacy.

The study also underscores the role of combination therapy, particularly pembrolizumab plus lenvatinib, which was associated with higher adverse drug reaction rates, most notably thyroid dysfunction in 60% of patients, similar to the study by Taylor et al., which showed 42% incidence of thyroid dysfunction, while maintaining favorable therapeutic responses [18]. These findings support the selective use of combination strategies in appropriately selected patients.

Limitations

Several limitations should be acknowledged. The inclusion of multiple malignancy types introduced heterogeneity in tumor biology, treatment response, and prognosis, potentially limiting subgroup-specific conclusions. Survival outcomes such as overall and progression-free survival were not evaluated. As this was a retrospective, single-center study, incomplete documentation of adverse drug reactions cannot be excluded. Additionally, small sample sizes in certain cancer subgroups may have limited statistical power, and prior or concomitant therapies could have influenced observed outcomes. Despite these limitations, this study provides valuable real-world evidence supporting the safety, tolerability, and clinical effectiveness of pembrolizumab across diverse malignancies, complementing data from controlled clinical trials.

## Conclusions

Pembrolizumab demonstrated meaningful clinical effectiveness across a diverse range of malignancies. Its therapeutic effectiveness was further enhanced when administered as part of combination regimens. Overall, Pembrolizumab exhibited a favorable safety profile, with adverse drug reactions being predominantly mild to moderate in severity and immune-related toxicities remaining largely manageable.

The combination of pembrolizumab and lenvatinib yielded encouraging clinical outcomes in cancer patients; however, this regimen was associated with an increased risk of thyroid dysfunction. Regular and timely monitoring of thyroid function is therefore essential during treatment to ensure early detection and appropriate management of endocrine adverse effects.

Taken together, these findings support pembrolizumab as an effective and generally well-tolerated therapeutic option across multiple cancer types. They also underscore the importance of appropriate patient selection, careful monitoring, and proactive management of adverse events to optimize treatment outcomes in real-world clinical practice.
